# New York Letter

**Published:** 1893-08

**Authors:** Wm. L. Russell

**Affiliations:** 101 West Ninety-Third Street; New York


### *CORRESPONDENCE.*

### OUR NEW YORK LETTER.

New York, July 12, 1893.

### CONTAGIOUS DISEASES.

The creation of a Department of Pathology, Bacteriology and Disinfection in our Municipal Health Department is a step forward in the management of contagious diseases. The organization and equipment of this department was described at a recent meeting of the Academy by Dr. H. M. Biggs, under whose direction it has been placed, and may prove of some value to your readers.

The management of cases of contagious disease in dwellings devolves upon a corps of medical inspectors and lay disinfectors. A medical inspector visits the infected apartments on the same day on which the notice has been received. Cards are immediately sent to the schools attended by the children residing in the house, or on the same floor if the disease be in a tenement. Where isolation of the patient is not possible, he is removed. If otherwise, he is placed in a room with windows opening to the outside air, the furniture is removed and the doors to the hall sealed. Disinfectants will be supplied by the department, if necessary. A visit is made by the medical inspector every four days, and at the termination of the illness the rooms and contents are disinfected. This is accomplished by fumigation with sulphur fumes in the presence of moisture, and by washing or soaking with disinfectants all articles that will not be thereby injured. A complete disinfecting station has been established, to which bedding, carpets, clothing, etc., are taken, and exposed to sulphur fumes and superheated steam under pressure. Wagons of different colors are provided for conveying and returning these articles, so as to avoid any possibility of confusion. The employees who handle the articles before and after disinfection also wear different uniforms. The disinfecting chamber is placed in the center of the station, and the portions of the building on each side of it are entirely disconnected. The articles are thus brought by one set of employees in special wagons, are by them passed into the disinfecting chamber, from which they are taken by another set of employees, who convey them back to their owners in other wagons.

The pathological department has to do with the examination of bodies of suspected cases of cholera, typhus, and other contagious diseases. The bacteriological work of the department plays a more and more important role as the knowledge of micro-organisms increases. It is concerned principally with diagnosis, and with experimentation as to the relative value of disinfectants, and with examinations of water and food stuffs. An interesting feature of this department is in the investigation of diphtheria, which has been recently undertaken. It is now known that there is a true diphtheritic membrane, and a pseudo-diphtheritic membrane, which cannot be distinguished from the ordinary clinical methods. In the one case, however, the Klebs-Loefler bacillus is found, and in the other a micrococcus. Investigations made at the hospital for contagious diseases show that only those cases in which the bacillus is found are true diphtheria. The other cases are of much less importance. It is the intention of the department to make cultures in every case of suspected diphtheria, if requested to do so by the attending physician. Such examinations are of value in conserving the resources of the department, and in guiding the physician to the proper measures to insure the safety of the well members of the family.

The method of making these investigations consists in leaving at a number of drug stores, in different parts of the city, small cases containing the necessary materials. These cases contain two test tubes, in one of which is a sterilized cotton swab on a glass rod, and in the other a portion of blood serum. Any physician having a case of suspected diphtheria can obtain one of these cases, with a card directing him how to proceed. The cotton is to be rubbed over the exudation, and then over the surface of the blood serum. The tubes are then plugged with cotton stoppers and the cases returned to the drug stores. Within twenty-four hours a report will be received from the department. The facility with which these cultures can be made, and their practical value, is illustrated by an instance related by Dr. Brannan. The case was one of membranous tonsillitis occurring in his own child. He was much concerned regarding the safety of the other children, and as to the proper disposition to make of the case. He called to his assistance Dr. Park, the bacteriologist of the Health Department in charge of the diphtheria examinations. A culture was made, and next morning at breakfast time a report was received that the case was not true diphtheria.

In the discussion following Dr. Biggs' paper, Dr. Jacobi referred to the deplorable conditions in the tenement houses, where two rooms had to suffice for the accommodation of both the sick and the well. He also states a fact which is likely to be of interest to those who patronize hotels in this and other cities, viz.: that he had known diphtheria to occur year after year in the same suite of rooms in a fashionable hotel, which had never been properly disinfected in the intervals.

Dr. J. D. Byant, late health commissioner, gave an account of the poor facilities for dealing with contagious diseases in 1887 as compared with the present conditions described by Dr. Biggs. He stated that instead of the elaborate disinfecting station there was then nothing but a torpedo-shaped cooking table, and exposure to sulphur fumes was the only method of disinfection employed. He also spoke of the necessity for a hospital for private patients with contagious diseases, where proper privacy could be secured and where each patient could be attended by his own physician. As matters were now strangers at hotels and those living in boarding houses had no resource but to go to the Willard Parker Hospital or to North Brothers Island. The care given in these places was undoubtedly good, but it was such as was provided for the poorest in the city, and it was impossible to discriminate between individuals.

Dr. Sternberg, of the army, spoke of the difficulty of destroying. the bacillus of diphtheria when protected within particles of dried membrane. He drew attention also to the rôle played by domestic animals and insects in disseminating the germs of disease, the dejecta of flies having been found to contain cholera bacilli.

Dr. Roosa, the president, deplored the lack of attention given to the provision of a hospital for contagious ophthalmia. These cases were now received in the general ophthalmic hospitals, a procedure which was highly improper.

In closing Dr. Biggs mentioned the interesting fact that of the twelve cholera cases occurring in this city last year, all but two or three were persons connected with food trades. Seven or eight belonged to butchers' families.

In answer to an inquiry regarding the efficiency of sulphur fumes as a disinfectant, he said that it was only moderate and then only in the presence of moisture. Three pounds of sulphur to each thousand cubic feet should be burned, and the room must be absolutely closed.

### INSTRUCTION IN CONTAGIOUS DISEASES.

A plan to provide bedside instruction in these diseases was suggested at a recent meeting of the academy by Dr. J. W. Brannan. He stated that in Boston contagious diseases were received into the city hospital, and students were allowed opportunities for studying them. They contracted the diseases sometimes but had never carried them. The objections to be met were the fears of the public regarding the carrying of contagion, the danger to the students and possibly the sensitiveness of the patients. Students might be taught how to avoid danger, and in order to prevent them from conveying contagion, precautions might be taken such as the wearing of rubber gowns and hoods, and the careful disinfection of hands and hair.

As the hospital for contagious diseases was under the jurisdiction of the Board of Health, it would be impossible to introduce the professors from the different medical schools. The instruction, however, could be well given by the diagnosticians of the Health Board, those who were sent to examine suspected cases. These men should be paid by the colleges. The instruction should be at first limited to undergraduates of the third and fourth year, and practitioners might perhaps be received later.

Dr. Wm. H. Draper recognized the importance of giving instructions in these diseases, saying that many physicians failed to recognize them. He believed, however, that the best plan was to organize a fever hospital in the city, which should have its staff of attending physicians like the other hospitals. The difficulty in establishing such a hospital was the fear of the rich. The danger of contagion, he thought, was greatly exaggerated in the minds of both the laity and physicians. He stated that in King's College Hospital, London, diphtheria and scarlatina patients were placed in the several wards, no spread of these diseases having ever occurred.

Dr. Shrady said that though he had had an unusually extensive training in college, hospital and dispensary previous to beginning practice, he failed to recognize an ordinary case of measles which was one of his first cases.

Dr. H. M. Biggs said that he could promise the co-operation of the Health Department in the plan of instruction suggested by Dr. Brannan.

Dr. J. W. Roosevelt thought that fever wards should be attached to each of the general hospitals.

WM. L. RUSSELL.

101 *West Ninety-Third Street.*

